# The involvement of noradrenergic mechanisms in the suppressive effects of diazepam on the hypothalamic-pituitary-adrenal axis activity in female rats

**DOI:** 10.3325/cmj.2012.53.214

**Published:** 2012-06

**Authors:** Dubravka Švob Štrac, Dorotea Muck-Šeler, Nela Pivac

**Affiliations:** Division of Molecular Medicine, Ruđer Bošković Institute, Zagreb, Croatia

## Abstract

**Aim:**

To elucidate the involvement of noradrenergic system in the mechanism by which diazepam suppresses basal hypothalamic-pituitary-adrenal (HPA) axis activity.

**Methods:**

Plasma corticosterone and adrenocorticotropic hormone (ACTH) levels were determined in female rats treated with diazepam alone, as well as with diazepam in combination with clonidine (α_2_-adrenoreceptor agonist), yohimbine (α_2_-adrenoreceptor antagonist), alpha-methyl-*p*-tyrosine (α-MPT, an inhibitor of catecholamine synthesis), or reserpine (a catecholamine depleting drug) and yohimbine.

**Results:**

Diazepam administered in a dose of 2.0 mg/kg suppressed basal HPA axis activity, ie, decreased plasma corticosterone and ACTH levels. Pretreatment with clonidine or yohimbine failed to affect basal plasma corticosterone and ACTH concentrations, but abolished diazepam-induced inhibition of the HPA axis activity. Pretreatment with α-MPT, or with a combination of reserpine and yohimbine, increased plasma corticosterone and ACTH levels and prevented diazepam-induced inhibition of the HPA axis activity.

**Conclusion:**

The results suggest that α_2_-adrenoreceptors activity, as well as intact presynaptic noradrenergic function, are required for the suppressive effect of diazepam on the HPA axis activity.

Benzodiazepines are used for their anxiolytic, sedative-hypnotic, muscle relaxant, and anticonvulsant properties in the treatment of a variety of neuropsychiatric disorders (1,2), including anxiety and depression, which are often related to disturbances in the activity of hypothalamic-pituitary-adrenal (HPA) axis (3,4). Although these drugs exert most of their pharmacological effects via γ-aminobutyric acid_A_ (GABA_A_) receptors (5,6), benzodiazepine administration has been associated with alterations in neuroendocrine function both in experimental animals and humans (7-9). However, even after years of extensive studies, the complex mechanisms by which these widely used drugs produce their effects on the HPA axis are still not known.

Although most of the previous studies have demonstrated that classical benzodiazepines such as diazepam decrease the HPA axis activity in stressful contexts (10-14), under basal conditions they have been shown to stimulate (9,11,15-18), inhibit (15,19-22), and not affect (17,23-25) the HPA axis activity. Such diverse results might be related to several factors such as the dose and gender (15,16,20,21,26-28), or may also be a consequence of the net effect of non-selective benzodiazepines on the various GABA_A_ receptor isoforms (9).

Our previous studies demonstrated that while diazepam (1 mg/kg) produced no change in plasma corticosterone levels in male rats (15,20), it decreased basal levels of corticosterone in female rats (15,26). However, although diazepam inhibited the HPA axis activity of female rats following administration of lower doses (1 or 2 mg/kg) (15,20,21,26), it stimulated the HPA axis activity following administration of high doses (10 mg/kg) (15,16,26). Moreover, whereas the suppressive effect of the lower doses of diazepam (2.0 mg/kg) on the HPA axis activity in female rats involves the GABA_A_ receptor complex (21), increases in corticosterone levels by a higher dose of diazepam (10 mg/kg) do not involve the stimulation of GABA_A_ receptors (16). In addition, stimulatory effect of 10 mg/kg diazepam on the HPA axis activity in rats seems not to be mediated by the benzodiazepine/GABA/channel chloride complex or by peripheral benzodiazepine receptors, but rather by a cyclic adenosine monophosphate (AMP)-dependent mechanism (18).

Since our previous results suggested that the effect of a high dose of diazepam on the activity of the HPA axis in female rats might be due to a blockade of α_2_-adrenergic receptors (16), the aim of this study was to elucidate whether noradrenergic system also has a modulatory role in the inhibitory effect of 2.0 mg/kg diazepam on basal plasma adrenocorticotropic hormone (ACTH) and corticosterone levels in female rats.

## Methods

### Animals

Female adult Wistar rats (180-200 g), bred in our Institute, were housed under controlled conditions, with standard light-dark cycle and food and water freely available. The rats were caged in groups of five. Each experiment included approximately 30-35 rats and was performed twice. The total number of animals used in the study was 260. To minimize circadian variability of plasma ACTH and corticosterone levels, all experiments were performed between 8.00 and 12.00 am. The only exception was the experiment with reserpine in which reserpine was administered during the working and day-light time on the day prior to the experiment, so that the administration of yohimbine and diazepam, as well as sacrifice could be preformed until 12.00 am of the next day. The phases of the estrous cycle of female rats were not monitored or synchronized prior to experiments, since we presumed on the basis of previous studies (29-31) that the sexual cycle of female rats used in our study would not significantly affect diazepam-induced reductions of basal plasma corticosterone or ACTH levels. Before the experiments, all animals were adapted to handling and intraperitoneal (ip) injecting for 7 days. All animal care and experimental procedures were carried out in accordance with the National Institute of Health Guide for the Care and Use of Laboratory Animals (NIH publication No. 86-23, revised 1996), and with the Croatian law on animal welfare. Ethical approval was received from the Ministry of Agriculture – Veterinary Directorate and Ruđer Bošković Institute Ethics Review Board.

### Drugs

Diazepam (Krka, Novo Mesto, Slovenia) and clonidine (Boehringer Ingelheim on Rhein, Germany) were dissolved in 0.1 N HCl, followed by saline. Yohimbine (Sigma Chemical Co, St. Luis, MO, USA) and α-methyl-*p*-tyrosine (α-MPT, Sigma) were dissolved in distilled water. Reserpine (Sigma) was suspended in glacial acetic acid and 5.5% glucose was added to the final volume. All drugs and corresponding vehicles were prepared fresh, and after pH adjustment injected ip in a volume of 1 mL/100 g bw.

### Experimental procedure

Reserpine (10.0 mg/kg), alpha-methyl-*p*-tyrosine (400.0 mg/kg), yohimbine (0.5 and 3.0 mg/kg), clonidine (0.01 and 0.5 mg/kg), diazepam (2.0 mg/kg), or corresponding vehicles were injected ip 15h, 4h, 90-minute, 35-minute, and 30-minute prior to sacrifice, respectively. The doses and time points of the drugs used in the study were selected based on the previous studies conducted in our laboratory and those reported in literature. Namely, since the results of our dose-response study (data available on request) revealed that the most significant decrease in plasma corticosterone levels was produced by the administration of 2.0 mg/kg diazepam, this dose was further used in our experiments. The two doses of clonidine (0.01 and 0.5 mg/kg) were used with the aim to differentiate between presynaptic and postsynaptic effects of this drug (32,33). Yohimbine was administered in two doses (0.5 mg/kg and 3 mg/kg), which have been already used in various studies (34,35). In order to ensure the complete inhibition of tyrosine hydroxylase, the enzyme responsible for catecholamine synthesis, alpha-methyl-*p*-tyrosine (α-MPT) was administered in a high dose (400.0 mg/kg), previously reported to inhibit catecholamine synthesis (36). To find out whether diazepam affects the HPA axis activity in conditions which completely eliminate the noradrenergic transmission, rats were pretreated with yohimbine (3.0 mg/kg) in a combination with reserpine administered in a high dose (10 mg/kg), already used for catecholamine depletion by other authors (37). Animals were sacrificed by decapitation with a guillotine. Trunk blood was collected in prechilled tubes containing EDTA (1 mg/mL of blood), centrifuged (4°C, 10-minute, 1250 g) and plasma was stored at -20°C. Corticosterone levels were determined in plasma samples of 500 μL by a slight modification of the fluorometric method (21). ACTH levels were measured directly, without prior extraction, in a 200 µL of plasma, by a radioimmunoassay using commercially available ACTHK-PR kit (CIS bioindustries, Gif sur Yvette, France).

### Data analysis

Results were expressed as percents ± standard error of the mean of the values in control animals. Statistical evaluation of the results was done with GraphPad Prism version 4.00 (GraphPad Software, San Diego, CA, USA) by using one-way analysis of variance (ANOVA) followed by Tukey test. *P*-values of <0.05 were considered significant.

## Results

**Figure 1:**
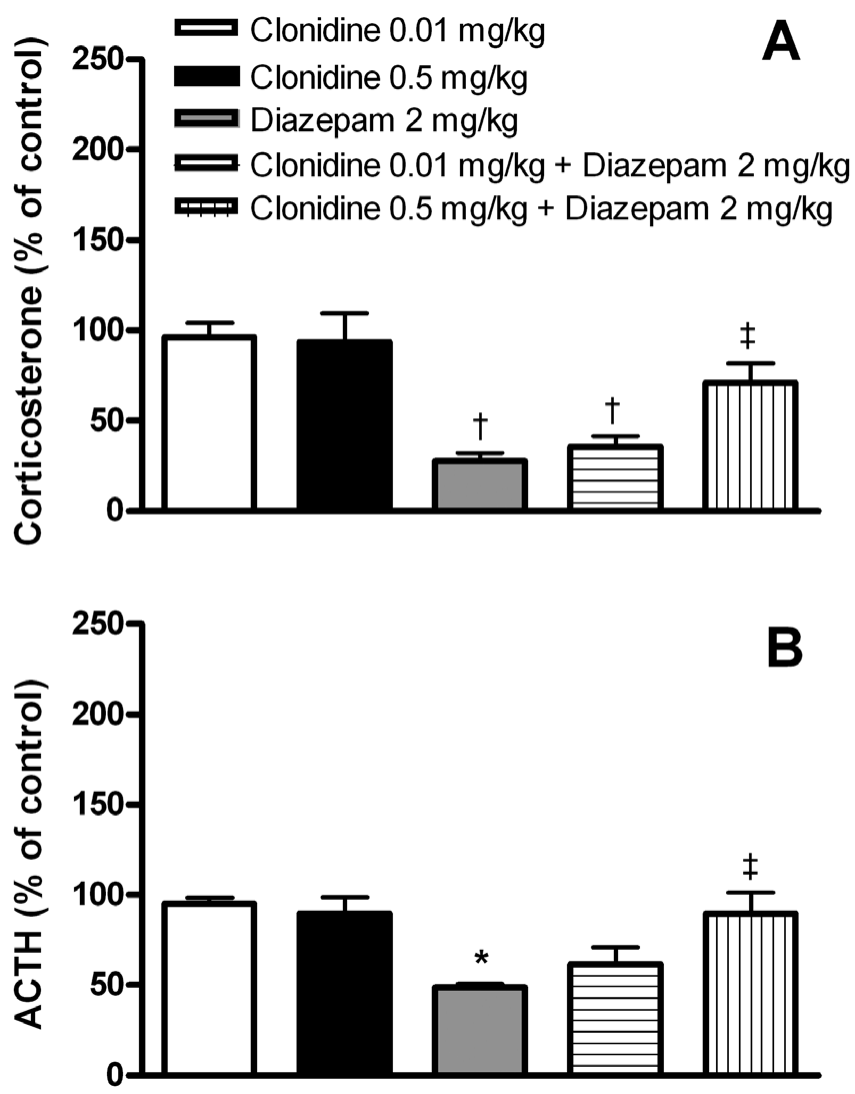
The effect of clonidine on diazepam-induced decrease of plasma corticosterone (**A**) and adrenocorticotropic hormone (ACTH) (**B**) concentrations. Clonidine (0.01 and 0.5 mg/kg) or its vehicle and diazepam (2.0 mg/kg) or its vehicle were injected ip 35 and 30 minutes, respectively, prior to sacrifice. The results are expressed as percents ± standard error of the mean of the values in control animals treated with the corresponding vehicles. The number of animals per group was 12 for determination of corticosterone levels and 7 for determination of ACTH levels. *P < 0.01; ^†^*P* < 0.001 vs the control vehicles-treated group; ^‡^*P* < 0.05 vs diazepam-treated group (one-way ANOVA followed by Tukey test).

**Figure 2:**
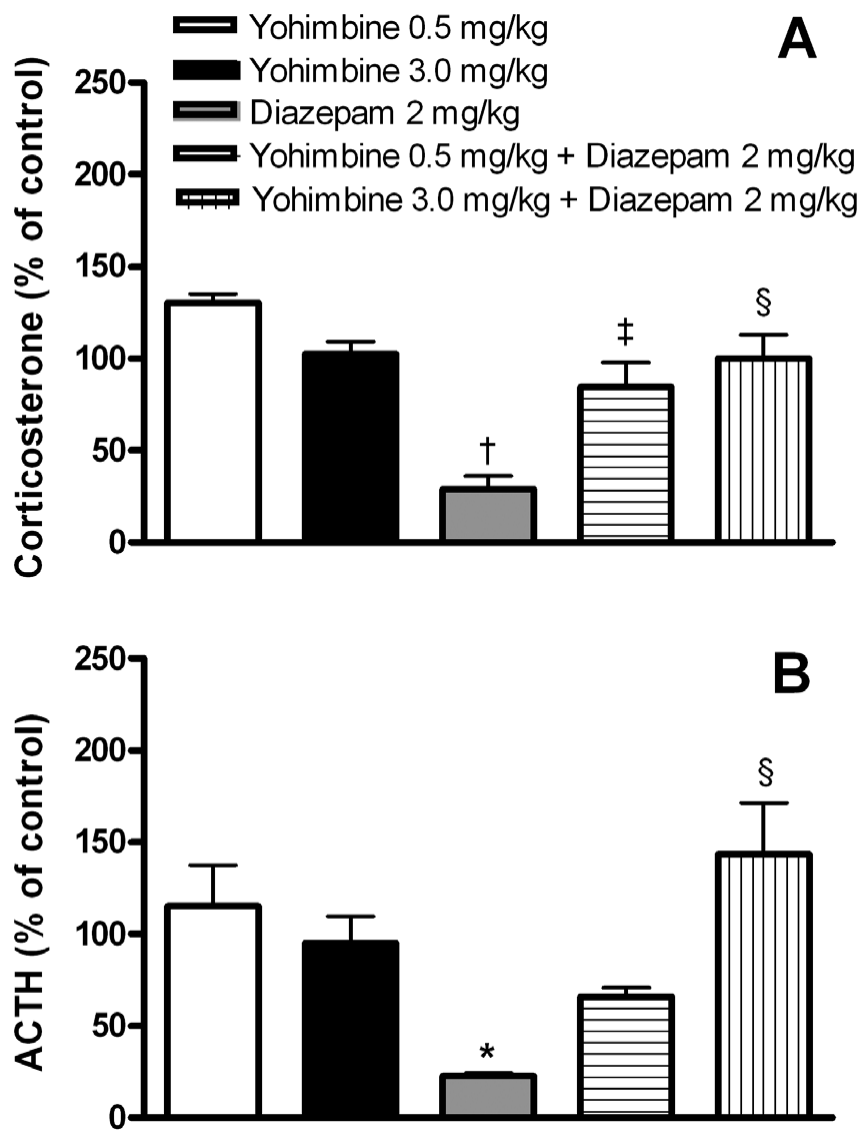
The effect of yohimbine on diazepam-induced decrease of plasma corticosterone (**A**) and adrenocorticotropic hormone (ACTH) (**B**) concentrations. Yohimbine (0.5 and 3.0 mg/kg) or its vehicle and diazepam (2.0 mg/kg) or its vehicle were injected ip 90 and 30 minutes, respectively, prior to sacrifice. The results are expressed as percents ± standard error of the mean of the values in control animals treated with the corresponding vehicles. The number of animals per group was 12 for determination of corticosterone levels and 7 for determination of ACTH levels. *P < 0.01; ^†^*P* < 0.001 vs the control vehicles-treated group; ^‡^*P* < 0.01; ^§^*P* < 0.001 vs diazepam-treated group (one-way ANOVA followed by Tukey test).

**Figure 3:**
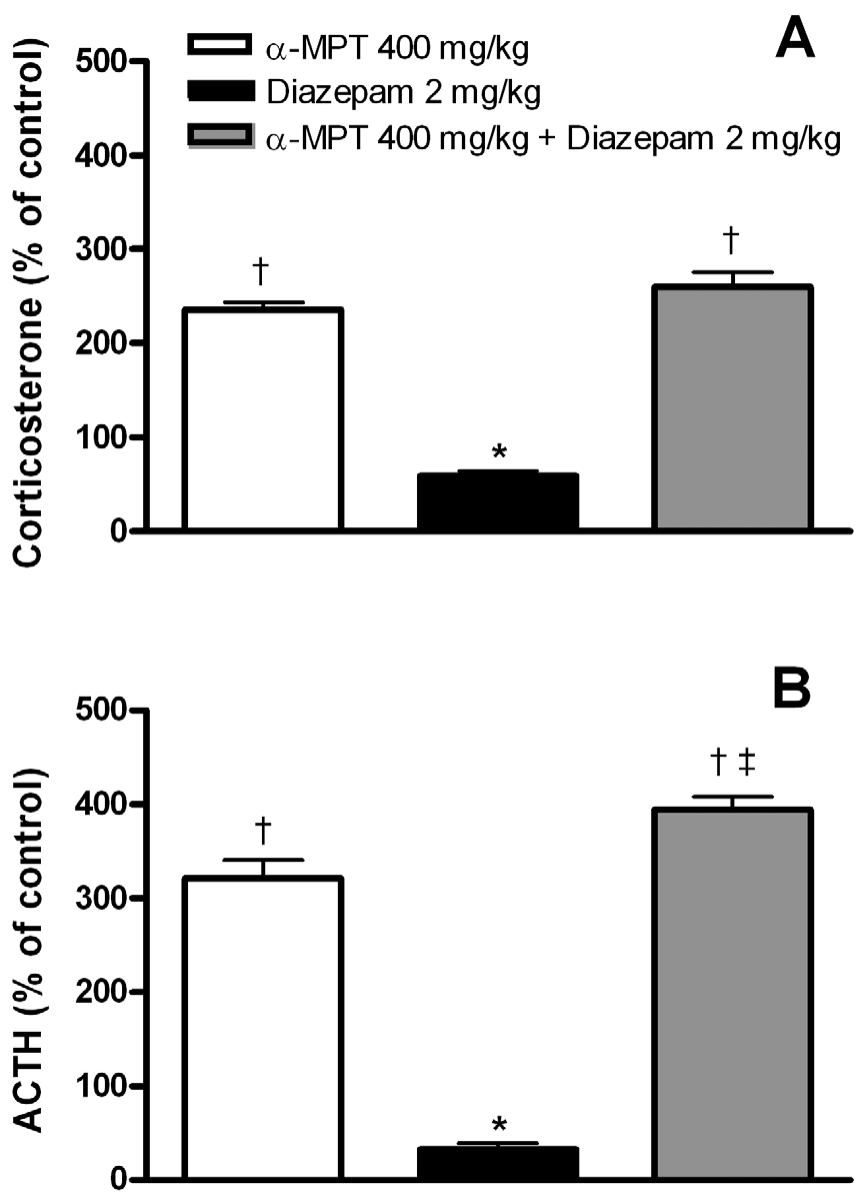
The effect of alpha-methyl-*p*-tyrosine (α-MPT) on diazepam-induced decrease of plasma corticosterone (**A**) and adrenocorticotropic hormone (ACTH) (**B**) concentrations. Alpha-methyl-*p*-tyrosine (400.0 mg/kg) or its vehicle and diazepam (2.0 mg/kg) or its vehicle were injected ip 240 and 30 minutes, respectively, prior to sacrifice. The results are expressed as percents ± standard error of the mean of the values in control animals treated with the corresponding vehicles. The number of animals per group was 15 for determination of corticosterone levels and 9 for determination of ACTH levels. *P < 0.01 or *P* < 0.05 vs the control vehicles-treated group; ^†^*P* < 0.001 vs the control vehicles-treated and diazepam-treated group; ^‡^*P* < 0.01 vs alpha-methyl-*p*-tyrosine-treated group (one-way ANOVA followed by Tukey test).

**Figure 4:**
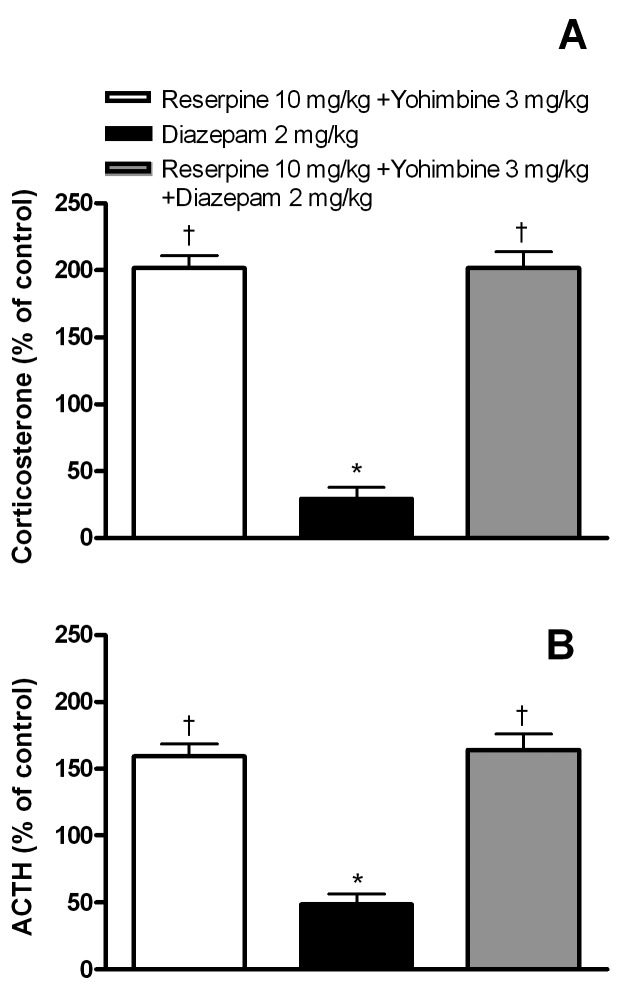
The effect of combined pretreatment with reserpine and yohimbine on diazepam-induced decrease of plasma corticosterone (**A**) and adrenocorticotropic hormone (ACTH) (**B**) concentrations. Reserpine (10.0 mg/kg) or its vehicle, yohimbine (3.0 mg/kg) or its vehicle, and diazepam (2.0 mg/kg) or its vehicle were injected ip 15 hours, 90 minutes, and 30 minutes, respectively, prior to sacrifice. The results are expressed as percents ± standard error of the mean of the values in control animals treated with the corresponding vehicles. The number of animals per group was 14 for determination of corticosterone levels and 7 for determination of ACTH levels. *P < 0.01 vs the control vehicles-treated group; ^†^*P* < 0.001 vs the control vehicles-treated and diazepam-treated group (one-way ANOVA followed by Tukey test).

The administration of 2.0 mg/kg of diazepam to female rats significantly reduced plasma corticosterone (*P* < 0.001) and ACTH (*P* < 0.01) levels. Both doses of clonidine (0.01 and 0.5 mg/kg) failed to affect plasma corticosterone and ACTH levels in control rats, but clonidine administered in a dose of 0.5 mg/kg antagonized diazepam-induced decrease of both hormones (*P* < 0.05) (Figure 1A and 1B). The administration of yohimbine in two doses (0.5 mg/kg and 3 mg/kg) elicited no effect on basal plasma corticosterone and ACTH levels. Although diazepam-induced reduction of plasma corticosterone levels (*P* < 0.001) was reversed already with 0.5 mg/kg yohimbine (*P* < 0.01) (Figure 2A), the higher (3.0 mg/kg) dose of yohimbine was required to counteract (*P* < 0.001) the effect of diazepam (*P* < 0.01) on plasma ACTH levels (Figure 2B). The administration of α-MPT significantly increased (*P* < 0.001) plasma corticosterone and ACTH levels (Figure 3A and 3B). Although diazepam significantly decreased (*P* < 0.001) plasma corticosterone and ACTH levels in control rats, it was unable to diminish the increase of corticosterone and ACTH in the rats pretreated with α-MPT (Figure 3A and 3B). Plasma corticosterone levels were similar between rats treated with α-MPT alone or α-MPT in combination with diazepam, but the combination of α-MPT and diazepam had an additive effect on plasma ACTH levels (*P* < 0.01). The combined treatment with reserpine and yohimbine induced a significant increase (*P* < 0.001) in plasma corticosterone and ACTH levels (Figure 4A and 4B). Diazepam significantly decreased plasma corticosterone and ACTH levels in control rats (*P* < 0.01), but could not suppress the elevated concentrations of both hormones in the rats pretreated with reserpine and yohimbine (Figure 4A and 4B).

## Discussion

The observed inhibitory effect of 2.0 mg/kg diazepam on the HPA axis activity agrees with our previous studies that used the same (21) or similar (20,26) doses and timing of diazepam administration, but disagrees with some results which demonstrated no changes in the levels of corticosterone induced by diazepam treatment (23). Some of the discrepancies considering the effects of diazepam on the basal HPA activity (stimulation, inhibition, or no effect) could be ascribed to the differences in sex (15,20,28,38), age (13,39), basal vs stressful conditions (10,13,38), or the strain of the animals used (40).

The lack of an effect of clonidine on plasma corticosterone and ACTH levels observed in our study agrees with other data (41,42). The finding that pretreatment with clonidine counteracted the diazepam-induced suppression of corticosterone and ACTH levels suggested that α_2_-adrenoreceptors are probably involved in the inhibition of the HPA axis produced by diazepam. However, yohimbine also reversed the inhibitory effect of diazepam on plasma corticosterone and ACTH levels, and these results are in line with a study showing that yohimbine prevented decreases in both of these hormones induced by the intraventricular administration of GABA (43).

The fact that treatments with α_2_-adrenoreceptor agonist clonidine and α_2_-adrenoreceptor antagonist yohimbine had the same effects, ie, that both abolished diazepam-induced inhibition of the HPA axis activity, might be explained by the pre- vs post-synaptic activities of these drugs. Namely, while the effect of clonidine administered in a dose of 0.5 mg/kg is likely to be produced by activation of postsynaptic α_2_-adrenoceptors (32), the observed effect of yohimbine is probably due to the blockade of inhibitory presynaptic α_2_-adrenoceptors, resulting in increased noradrenaline release (44). However, as the action of clonidine and yohimbine could be mediated by the activation or by the blockade of α_2_-adrenoreceptors (32,41,45), and as the drugs used are not absolutely selective, other more receptor-distinctive agonists and/or antagonists might be useful in revealing the potential involvement of these receptors in diazepam-induced suppression of the HPA axis.

The results showing that diazepam was unable to counteract the enhancement of corticosterone and ACTH levels elicited by α-MPT (an inhibitor of tyrosine hydroxylase, the enzyme responsible for catecholamine synthesis), suggested that diazepam suppresses the HPA axis activity by an action that also requires the intact presynaptic noradrenergic function. Moreover, treatment with combination of reserpine (a catecholamine depleting drug) and yohimbine (a α_2_-adrenoreceptor antagonist), which creates a condition that diminishes noradrenergic transmission, completely abolished the inhibitory effect of diazepam on plasma concentrations of corticosterone and ACTH. These results indicate that diazepam cannot exert any inhibitory influence when HPA axis is stimulated by drugs affecting noradrenergic neurotransmission such as α-MPT and reserpine, already reported to enhance corticosterone and/or ACTH levels (41,46,47).

**Figure 5:**
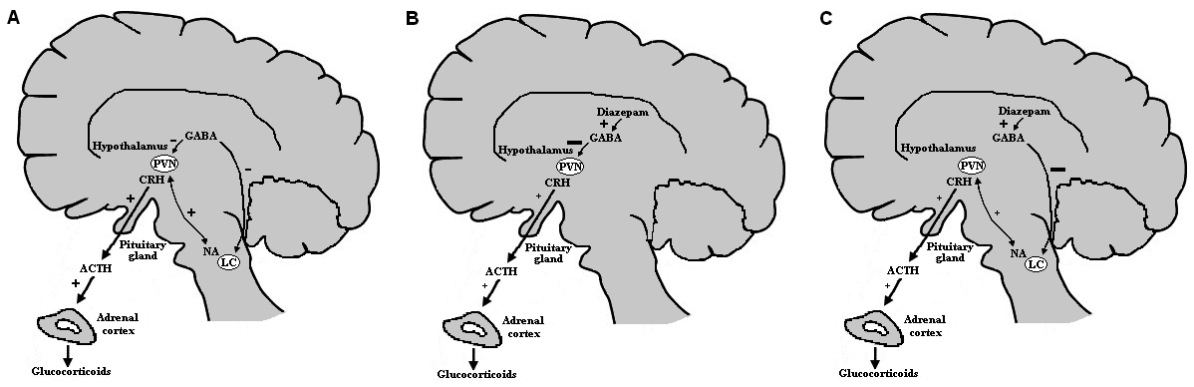
Hypothetical mechanisms involved in the inhibitory effect of diazepam on the hypothalamic-pituitary-adrenal (HPA) axis activity – modified from Carrasco et al (12). The γ-amino-butyric acid (GABA) system is involved in the regulation of the HPA activity via inhibition of corticotrophin-releasing hormone (CRH) and noradrenergic neurons (**A**). The diazepam-enhanced GABAergic neurotransmission results in the increased inhibition of CRH neurons in the hypothalamic paraventricular nucleus (PVN) and consequently in the decrease of plasma adrenocorticotropic hormone (ACTH) and glucocorticoid levels (**B**). However, this inhibitory effect of diazepam could be achieved, besides direct GABAergic inputs to CRH neurons, also by the interaction of GABAergic neurons from the surrounding hypothalamic regions and noradrenergic neurons in the locus coeruleus (LC), which also exhibit reciprocal neural connections with CRH neurons in PVN (**C**). This hypothesis is supported by our results demonstrating that intact noradrenergic function is essential for the diazepam-induced suppression of the HPA axis activity, while activation of postsynaptic or blockade of presynaptic α_2_-noradrenergic receptors reduces inhibitory effect of diazepam.

To our knowledge, the present study was the first to demonstrate that besides central benzodiazepine receptors, the part of GABA_A_ receptor complex (21), α_2_-adrenoreceptors activity, and intact presynaptic noradrenergic function were required for the suppressive effect of 2.0 mg/kg diazepam on the activity of the HPA axis in female rats. The suggestion that diazepam affects the HPA axis via noradrenergic system (Figure 5) also agrees with the previously shown interactions between diazepam and noradrenergic function (48-50).

However, the fact that diazepam (1 mg/kg) produced no change in basal plasma corticosterone levels in male rats (15,20) points out differences in the regulatory mechanisms of the HPA axis activity between males and females (10,30,38,51,52). These findings are in line with the studies demonstrating that the effects of benzodiazepine exposure on CRH system, including corticosterone and ACTH levels, are influenced by gender-related factors (27,30). Namely, gonadal steroid hormones including estrogens and androgens have been shown to modulate the GABA/BZ receptors or their responses, as well as the HPA axis activity implicated in the different stress responses between genders (27,30,53,54). Moreover, sexually dimorphic alternations in CRH system and HPA responses to stress are suggested to play a role in the etiology of affective and anxiety-related disorders and may be affected by both estrogen and progesterone (27).

Such neuropsychiatric conditions have been also associated with perturbations of endogenous neurosteroids within the CNS (55,56). Neurosteroid levels are dynamically regulated in response to a number of physiological states, such as stress, development, puberty, pregnancy, menopause, and during the ovarian cycle (57,58). In addition, it has been demonstrated that treatment with certain psychoactive drugs can affect neurosteroid content in the brain (59,60). Namely, certain benzodiazepines including diazepam, besides enhancing the GABA_A_ receptor function, have been shown to promote the synthesis of neurosteroids (61,62). Hence, in agreement with the known role of neurosteroids as potent positive allosteric modulators of GABA action at GABA_A_ receptors (62), the effects of diazepam might be potentiated by endogenous neurosteroids (63). As neurosteroids participate in the homeostatic regulation of HPA axis under basal as well as stress conditions (64), they could also contribute to diazepam-induced suppression of HPA axis activity, by additionally decreasing ACTH and corticosterone levels, especially during stress when rapid elevation of neurosteroid levels occurs (65,66).

In conclusion, our present and previous (16) data suggested that noradrenergic system might have an important modulatory role in the suppressive as well as in the stimulatory effects of diazepam on the HPA axis activity in female rats. As disturbances in the HPA axis activity are found in a variety of psychiatric disorders that are treated with benzodiazepines, including depression, insomnia, and anxiety (3,4), we could speculate that the therapeutic effects of benzodiazepines might be achieved not only by their well-known mechanisms, but also through modulation of the HPA axis activity via noradrenergic system. Considering the fact that mood and anxiety disorders as well as use of benzodiazepines are highly prevalent among women (28,67,68), these findings might be relevant for the therapeutic implications in female population. This might be particularly the case in the combined treatment of benzodiazepines and antidepressants, such as tricyclic antidepressants, noradrenaline reuptake inhibitors, and serotonin–noradrenaline reuptake inhibitors (69,70). These combinations are especially common in the treatment of co-morbid depression and anxiety. Since some antidepressant drugs might affect the noradrenergic neurotransmission, which seems to be involved in the opposite effects of different doses of diazepam on the HPA axis activity, a special attention should be given to adjustment of particular doses of benzodiazepines and antidepressants, when used in combination.
